# Probing Contaminant-Induced Alterations in Chlorophyll Fluorescence by AC-Dielectrophoresis-Based 2D-Algal Array

**DOI:** 10.3390/bios8010015

**Published:** 2018-02-11

**Authors:** Coralie Siebman, Orlin D. Velev, Vera I. Slaveykova

**Affiliations:** 1 Environmental Biogeochemistry and Ecotoxicology, Department F.-A. Forel for Environmental and Aquatic Sciences, Earth and Environmental Science, Faculty of Science, University of Geneva, 66 Boulevard Carl-Vogt, CH-1211 Geneva 4, Switzerland; coralie.siebman@gmail.com; 2 Department of Chemical and Biomolecular Engineering, North Carolina State University, Raleigh, NC 27695-7905, USA; odvelev@ncsu.edu

**Keywords:** whole cell array, dielectrophoresis, flow cytometry, chlorophyll fluorescence, copper, copper oxide nanoparticles, mercury, *Chlamydomonas reinhardtii*

## Abstract

The investigation of contaminant impact on algae requires rapid and reliable cell collection and optical detection. The capability of alternative current (AC) dielectrophoresis (DEP) collection of whole cell arrays with combined fluorescence microscopy detection to follow the alterations of chlorophyll fluorescence during environmental contaminant exposure was explored. The application of an AC-field of 100 V cm^−1^, 100 Hz for 30 min to capture and immobilize the cells of green alga *Chlamydomonas reinhardtii* in two-dimensional (2D) arrays does not induce changes in chlorophyll fluorescence. The results demonstrate that DEP-based 2D-arrays allow non-invasive detection of chlorophyll fluorescence change upon exposure to high concentrations of copper oxide nanoparticles and ionic copper. These results were in agreement with data obtained by flow cytometry used as a comparative method. The tool was also applied to follow the effect of a number of ubiquitous contaminants such as inorganic mercury, methylmercury, and diuron. However, a statistically significant short-term effect was observed only for mercury. Overall, DEP-based 2D-arrays of algal cells with fluorescence detection appear to be suitable for stain-free probing the effects on the photosynthetic microorganisms in highly polluted environment.

## 1. Introduction

Among different biological responses used for ecotoxicological purposes, chlorophyll *a* fluorescence is widely applied for the determination of contaminant-induced effects on photosynthetic organisms [1,2]. Chlorophyll *a* fluorescence measurements offer a possibility for a rapid, sensitive, and non-intrusive [3,4] investigation of the photosynthesis process [4]. Chlorophyll fluorescence can be thus used as a suitable toxicity indicator and can complement routine bioassays such as the ones based on growth inhibition [2,4]. Some of the most hazardous contaminants present in the environment can induce a modification in photosynthesis activity [4,5] and thus an alteration of the fluorescence of chlorophyll in a concentration-dependent manner [4,5]. A number of studies have already demonstrated the effect of different contaminants on the photosystem II (PSII) of the photosynthetic organisms [3]. The interaction of these contaminants, from herbicides or pesticides to heavy metals, with PSII could cause an inhibition of the electron transfer from the primary acceptor QA to the secondary quinone QB along the photosynthesis chain [4,5,6] and cause variation of the fluorescence from PSII which can be monitored [4,5].

During the past few years, several methods were developed to assess the variation in chlorophyll fluorescence. The inhibition by contaminants of the electron transport in PSII inducing a decrease in the fluorescence of chlorophyll can, for example, be monitored by flow cytometry (FCM) [7]. This technique allows rapid measurements of single cells without any pre-treatment or pre-extraction and gives simultaneous information on several parameters, such as cells volume or size and their fluorescent properties [8]. FCM is extensively used in the analyses of microalgae from aquatic environment and is considered as a validated alternative to more traditional methods for stress effect determination [7,8,9]. For example, the changes in chlorophyll fluorescence of *Chlamydomonas eugametos* cells exposed to paraquat, a herbicide, or in *Phaeodactylum tricornutum* exposed to copper [9] and in *Chlamydomonas reinhardtii* exposed to copper and copper oxide nanoparticles (CuO-NPs) were followed by FCM. In both cases, FCM was revealed as a useful tool in toxicity assessment using microalgae and different contaminants.

The development of new tools could improve the fluorescent detection in a rapid and non-invasive way. Very recently, optical biosensors for the detection of the effect of cadmium(II), lead(II), and anthracene on the chlorophyll fluorescence intensity of *Anabaena flosaquae*, *Chlorella vulgaris*, and *Euglena gracilis* microalgae in suspension were developed via the encapsulation of microalgae in sol–gel matrices [10]. Nevertheless, real-time monitoring using optical biosensors is understudied and most of the measurements involve electrochemical biosensors [11]. However, this kind of biosensors have some drawbacks such as electrode fouling, lack of stability and poor selectivity of the analyte [12]. Nevertheless, new optical sensors were recently developed for dynamic analyzes including a portable oxidative stress sensor allowing the detection of ROS release by the microalga *C. reinhardtii* exposed to different nanoparticles [13]. In this context, the present study explores the capabilities of the newly developed 2D-microalgal arrays to follow the change in the chlorophyll fluorescence during the short-term exposure to an emerging contaminant such as copper oxide nanoparticles (CuO-NPs), as well as other contaminants including dissolved Cu, inorganic mercury (Hg), methylmercury (MeHg), and diuron. This device uses alternative current (AC) dielectrophoresis (DEP) for rapid on-chip cell trapping and concentration of green microalga *C. reinhardtii* [14] combined with fluorescent detection. To our knowledge, only few studies had combined the key technique of DEP cells manipulation with fluorescent detection. These few studies include manipulation of latex nanoparticles [15], BSA [16], and more recently the study involving *C. reinhardtii* in which a DEP-based biosensor was developed and applied for ROS detection [17].

## 2. Materials and Methods

### 2.1. Algal Cell Cultures and Test Media

A unicellular green alga *C. reinhardtii* (CPCC 11, Canadian Phycological Culture Centre, Waterloo, ON, Canada), was grown in a 4× diluted Tris-Acetate-Phosphate medium (Sigma-Aldrich, Buchs, Switzerland) to the mid-exponential growth phase at 20 °C under continuous illumination of 6000 lux and continuous shaking at 115 rpm (INFORS HT, Basel, Switzerland). The cells were centrifuged at 3000 rpm for 5 min (Omnifuge 2.0 RS, Heraeus Sepatech GmbH, Osterode/Harz, Germany), the supernatant was removed, and the remained cells were suspended in the test medium. The final concentration of cells was fixed at 5 × 10^6^ cells mL^−1^. The test medium was composed of 10^−4^ M 3-(N-Morpholino)propanesulfonic acid (MOPS) (Sigma-Aldrich, Buchs, Switzerland) with or without the addition of various contaminants. Standard stock solutions of 0.1 M copper sulfate (Cu), 1 mg L^–1^ methylmercury (MeHg) in 12% nitric acid and diuron were purchased from Sigma-Aldrich. 10^−3^ M diuron stock solution was filtered through 0.45 µm-pore size filters (Millipore, Billerica, MA, USA). The stock dispersion of 2 g L^−1^ of copper oxide nanoparticles of 99.1% purity (Nanostructured and Amorphous Materials, Houston, TX, USA) was prepared in MilliQ water and sonicated at 130 W, 20 KHz for 1 min before handling (Sonics Vibra Cell, Sonic and Materials, Newtown, CT, USA). The average hydrodynamic size and zeta potential of 241 ± 13 nm and −22.2 ± 0.6 mV for CuO-NPs in 10^−4^ M MOPS were determined by Zetasizer Nano-ZS (Malvern, Renens, Switzerland). Less than 10% of dissolved Cu into the CuO-NPs suspensions was found by inductively coupled plasma mass spectrometry after dialysis (1000 Da cut-off of the membrane) [17].

### 2.2. Measurements of Chlorophyll Fluorescence by 2D Arrays Combined with Fluorescence Microscopy

**Figure 1:**
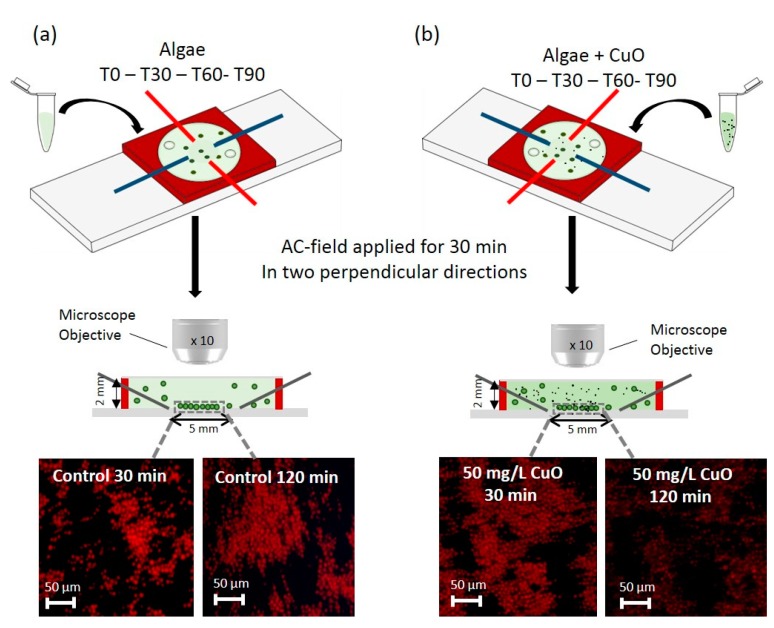
Experimental set-up and plan (**a**) cells in suspension in the absence of contaminant (control) and (**b**) in the presence of CuO-NPs, as an example. The samples are injected into the chamber after 0, 30, 60, and 90 min of exposure in the medium without or with contaminant (T0-T30-T60-T90). Four-point needle electrodes are used for a formation of 2D-cell assembly (upper panel). The electric field was applied to one pair of electrodes and was switched to the other pair in perpendicular direction every 2 min allowing the formation of planar 2D-cell assembly which enables convenient microscopy analysis without out-of focus cell background. The cells and media are confined in a microfluidic chamber of silicone rubber (red) with optically transparent top. Transverse section of the microfluidic chamber (middle panel) illustrates the 2D-assembly of cells formed in the 5 mm-gap of the four point needle electrodes. The 2D-arrays corresponding to different exposure times (T + 30 min), are observed under 10× magnification objective using fluorescence channel DAPI after 30 min of AC-field application at 50 V and 100 Hz. The chamber and the cell size are not to scale.

The assembly of algal cells in 2D arrays was performed with 5 mm gap four-point needle electrodes as detailed in our previous work [17]. The suspension of *C. reinhardtii* in test medium was injected into the chamber and the AC-field was then applied in two perpendicular directions for 2 min each. When the AC-field is applied, first 1D-cell chains are formed in the direction of the electric field due to the directional attractive dipolar interactions between the cells. The AC-field was then switched to the other electrode pair to let the cells realign along the perpendicular direction. The switching process was repeated every 2 min for a total duration of 30 min until the cells formed two dimensional structures (Figure 1). No formation of the cellular stacks was observed, which increases the data accuracy as all of the observed and measured cells are positioned precisely in the focal plane of the microscope. Optimization of the conditions for the DEP assembly of 2D-array of cells was performed in our previous study [17]. Briefly, the algal cells are subjected to non-uniform AC electric fields and form 2D-membranes using positive DEP near the bottom of the chamber, thus each cell is effectively trapped by the combination of the field and close packing in the 2D-assembly. The observation of the cell array was performed in the central area of the chamber to minimize artifacts arising from AC-electroosmotic (ACEO) phenomena near the electrode tips.

To confirm the absence of the effect of AC-field on the fluorescence of *C. reinhardtii*, the chlorophyll fluorescence was detected in the experimental setup with and without applied AC-field. The 2D-arrays formed by DEP were observed with fluorescence microscopy (BX61, OLYMPUS, Volketswil, Switzerland) using a digital camera (XC30, OLYMPUS, Volketswil, Switzerland) connected to the Cellsens software (Cellsens dimension OLYMPUS, Volketswil, Switzerland). For each experiment, images were collected at 30, 60, 90, and 120 min exposure in DAPI fluorescence channel with excitation and emission wavelength of 320 to 390 nm and 430 to 490 nm respectively (Figure 1).

Chlorophyll fluorescence of 2D arrays of *C. reinhardtii* was measured and the corrected fluorescence (*CF*) was determined following Equation (1) [18]:

(1) $$CF_{obj}=\;\overset{i=N_{obj}}{\underset{i=1}{\sum }}F_{obj\;i}-N_{obj}\;\frac{\sum_{J=1}^{J=N_{bkg}}F_{bkg\;j}}{N_{bkg}},$$

where *CF* is the corrected fluorescence; *F* is the fluorescence intensity in the object *i* and in the background *j* and measured at each pixel, *obj* is the 2D-cellular array known as the object of interest, *bkg* corresponds to the background and *N* is the number of pixel in the 2D-cellular array or in the background [18]. Based on this equation, the fluorescence of out-of-focus background of each obtained image was subtracted from the fluorescence of on-focus 2D-cellular array. Mean *CF* value per pixel was then obtained by normalizing the *CF* value by the area of the 2D-algal array. The images obtained from microscopy were treated with ImageJ (National Institute of Mental Health, Bethesda, MD, USA). The calculation and analysis were processed on 8-bit images with ImageJ.

### 2.3. Measurements of Chlorophyll Fluorescence by Flow Cytometry

Chlorophyll fluorescence of *C. reinhardtii* was followed in parallel with FCM. FCM analyses were performed using a BD Accuri C6 flow cytometer (BD Biosciences, San Jose, CA, USA) with an Accuri CSampler (BD Biosciences, San Jose, CA, USA). The data such as the number of counted cells and chlorophyll fluorescence from red channel (670 nm) when excited with 488 nm argon laser were acquired and analyzed using the BD Accuri C6 Software 264.15 (BD Biosciences, San Jose, CA, USA). Data were collected to 10,000 events for each sample after 30, 60, 90, and 120 min exposure to contaminants.

### 2.4. Chlorophyll Fluorescence Changes upon Contaminant Exposure

To evaluate the impact of CuO-NPs and Cu, Hg, and MeHg as well as diuron on chlorophyll fluorescence, *C. reinhardtii* was exposed for 2 h to several concentrations of these micro-contaminants: 50 and 10 mg L^–1^ CuO-NPs, 10^−5^ M and 10^−6^ M Cu, 10^−7^ M and 10^−8^ M Hg. 10^−7^ M and 10^−9^ M MeHg and 10^−6^ M and 10^−7^ M diuron. The different concentrations of contaminants were chosen based on the previous results for oxidative stress in *C. reinhardtii* obtained by 2D-algal array [17]. The algal suspension of 5 × 10^6^ cells mL^−1^ was exposed to each contaminant for 30, 60, 90, and 120 min. To probe the use of the 2D-arrays combined with fluorescence measurements for a rapid detection of the algal response to contaminants we have focused only on short-term exposure. For each exposure time, the suspension was injected into the microfluidic chamber 30 min prior to the observation time and AC-field was applied for 30 min (Figure 1). Algae suspended in 10^−4^ M MOPS in the absence of contaminants was used as a control. Each exposure experiment was repeated three times.

### 2.5. Statistical Treatments

Statistical differences within chlorophyll fluorescence under different treatments (e.g., DEP parameters, contaminants) were evaluated using the Student–Newman–Keuls test for multiple comparisons in Sigma Plot 11 (Systat Software Inc., San Jose, CA, USA). All data obtained in the presence of contaminants were normalized by the negative control.

## 3. Results and Discussion

### 3.1. Effect of DEP on Chlorophyll Fluorescence

**Figure 2:**
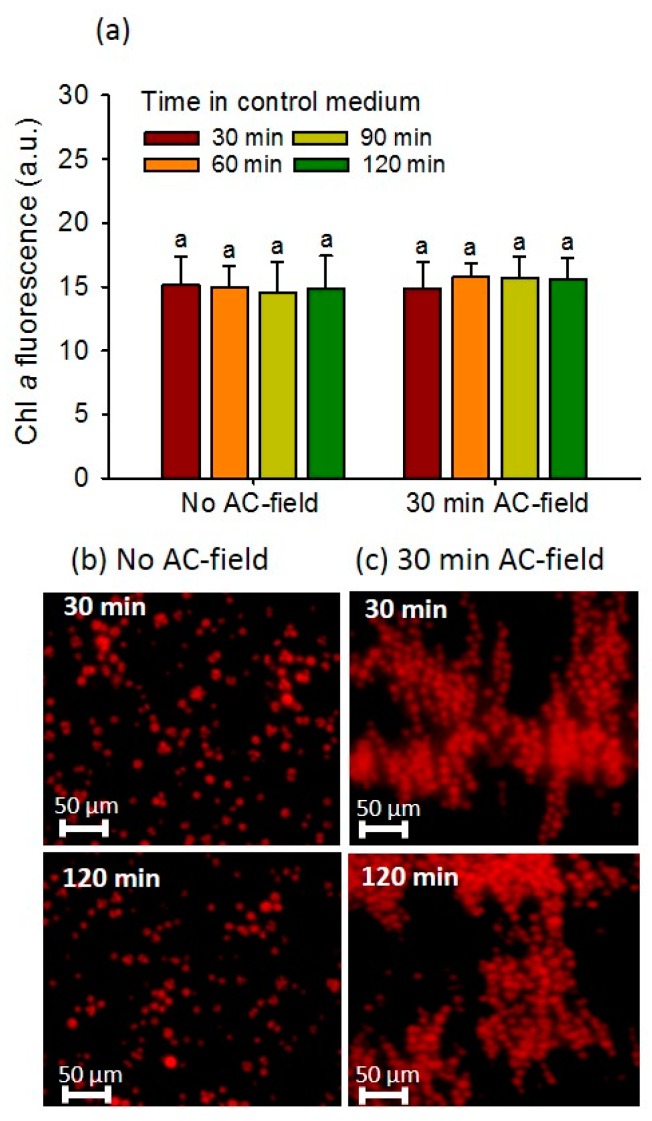
Effect of the AC-field duration on chlorophyll fluorescence intensity of 2D-cellular arrays. (**a**) Fluorescence intensity corresponding to the mean corrected fluorescence intensity (*CF*) per pixel calculated from the fluorescence images obtained in DAPI channel with or without the application of AC-field. The letter ‘a’ indicates the non-statistically significant differences and were obtained by Student–Newman–Keuls test with *p* < 0.05. Microscopy images of autofluorescent 2D-arrays with (**b**) no AC-field and (**c**) AC-field applied for 30 min. The initial concentration of algae in suspensions was 5 × 10^6^ cells mL^−1^.

The advantage of making single-layer, DEP-trapped, and close-packed layer without 3D- stacking is that it allows facile and reliable microscopy analysis, by capturing and aligning the cells in the microscope focal plane [17]. As the purpose of the cell array is its use for environmental sensing of contaminant effects on green microalgae, the DEP cell manipulation should not induce alteration in the measured algal characteristics such as chlorophyll fluorescence. No effect of the DEP manipulation for 2D-array formation on the algal fluorescence was observed (Figure 2), as revealed by the comparable fluorescence intensity of the cells with or without an applied electric field while the cells were incubated from 30 min to 120 min in the absence of contaminant. Indeed, the chlorophyll fluorescence intensity of the 2D-array remained unchanged when AC-field was applied for 30 min as compared with the fluorescence of the cells without AC-field (Figure 2a). Microscopy images show comparable bright red fluorescence corresponding to chlorophyll for the systems with no AC-field applied and AC-field applied for 30 min independently of the incubation time from 30 to 120 min (Figure 2b,c). The results are consistent with literature reporting no effect of AC-field on the viability of green algae [14,17], yeast [19] and endothelial cells [20]. Although electric field with other parameters has been shown to affect the motion of the chloroplasts in the green alga *Eremosphaera* *viridis* [21], and thus could influence the detected fluorescence, no such phenomenon was observed after 30 min of AC-field application for *C. reinhardtii* in the present work.

### 3.2. Effect of CuO-NPs and Cu on Chlorophyll Fluorescence

**Figure 3:**
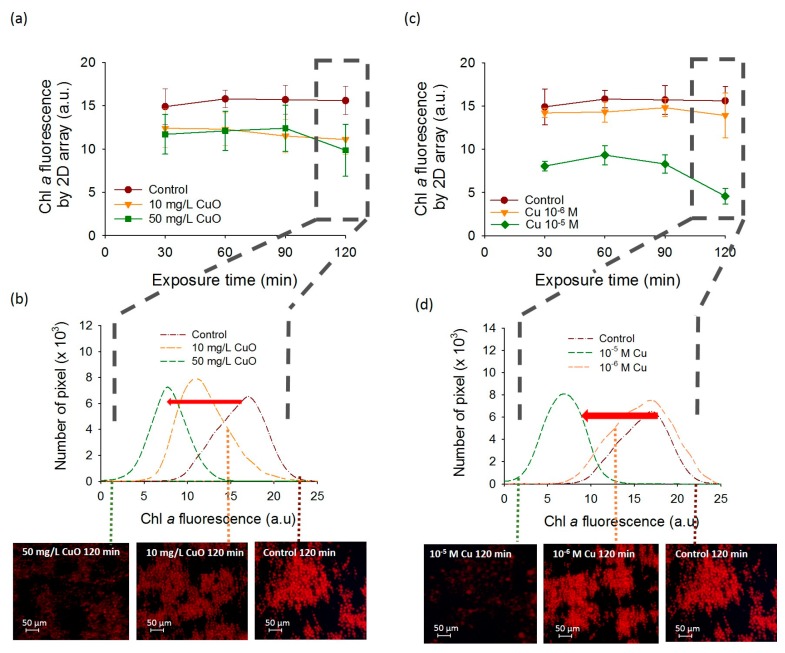
CuO-NPs and Cu-induced effects on the fluorescence of *C. reinhardtii* obtained by the 2D-array. Time course of the mean corrected fluorescence intensity (*CF*) per pixel of cells assembled in 2D-array by DEP exposed to increasing (**a**) CuO-NPs and (**c**) Cu concentrations. The data values are average and standard deviation from three replicates. Examples of the number of pixels obtained with the 2D-array versus corrected fluorescence of chlorophyll after 120 min of exposure time and illustrative microscopy images of chlorophyll fluorescence 2D-arrays with increasing (**b**) CuO-NPs and (**d**) Cu concentrations.

A significant decrease of chlorophyll fluorescence was observed for *C. reinhardtii* exposed to CuO-NPs as compared with the unexposed control algae. Indeed, after 120 min of CuO-NPs exposure time, the fluorescence obtained with 2D-array reached 15.6 ± 1.63 a.u. for the unexposed control while for 10 mg L^−1^ and 50 mg L^−1^ of CuO-NPs it reached 11.1 ± 1.76 a.u. and 9.86 ± 3.01 a.u. respectively (Figure 3a). However, at shorter exposure times, no significant differences in the fluorescence of algae exposed to 10 or 50 mg L^−1^ of CuO-NPs were found (Figure 3a,b). The shift in 2D-array histograms at 120 min also evidenced the significant decrease of the fluorescence in CuO-NPs exposed *C. reinhardtii* (Figure 3b), as well as a higher decrease of the fluorescence in the presence of 50 mg L^−1^ of CuO-NPs than with 10 mg L^−1^ of CuO-NPs. Since the effects of the CuO-NPs are considered to be at least partially driven by the dissolution [22,23], we also investigated the fluorescence of the cells exposed to dissolved copper over a period of 120 min. The effect of Cu 10^−5^ M on the chlorophyll fluorescence of the cells was more pronounced than that of CuO-NPs at 10 and 50 mg L^−1^ (Figure 3c,d). A decrease of the mean *CF* measured in DAPI channel corresponding to the algal fluorescence was observed with Cu compared with unexposed controls (Figure 3). Exposure to 10^−5^ M Cu resulted in a significant decrease of the algal fluorescence (bleaching). At this concentration, the decrease of fluorescence was already strong at 30 min exposure (8.05 ± 0.55 a.u.) and further decreased to 4.57 ± 0.91 a.u. after 120 min (Figure 3c,d). However, at 10^−6^ M Cu, no significant reduction of the mean *CF* was found from 30 to 120 min (Figure 3c,d). Indeed, no shift in 2D array pixel histograms at 120 min was observed at 10^−6^ M Cu compared to the control while 10^−5^ M Cu exposure induced an important shift in the direction of decrease of the fluorescence intensity.

**Figure 4:**
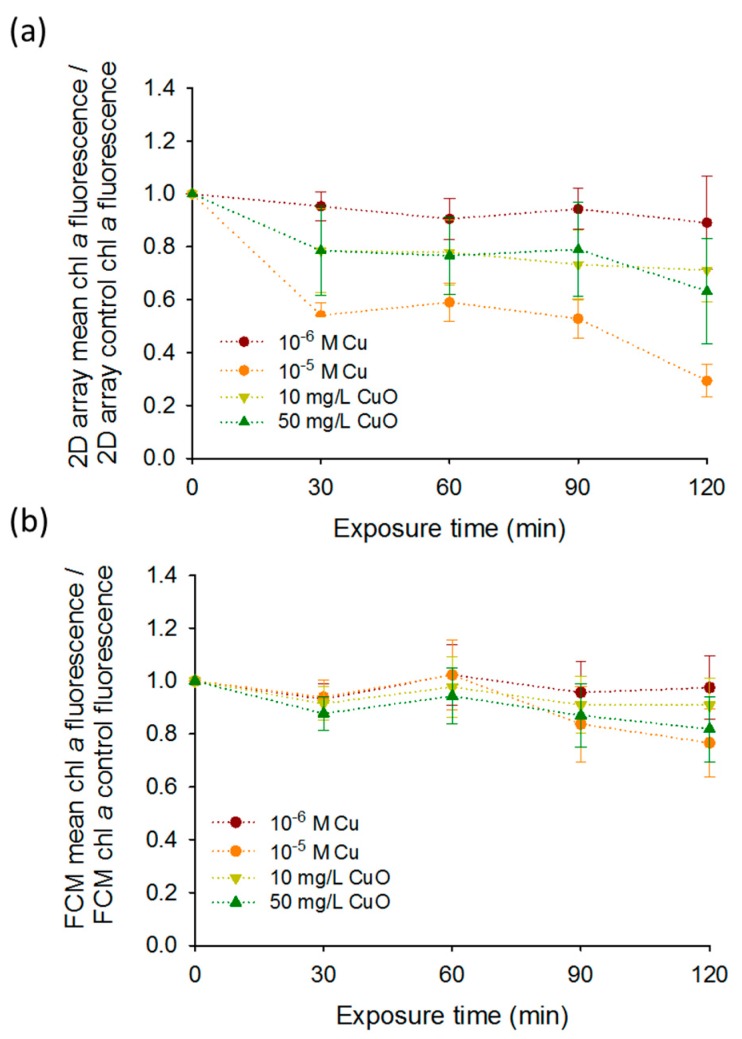
CuO-NPs and Cu effects on *C. reinhardtii* fluoresecence obtained by (**a**) 2D-array and (**b**) FCM. Time course of (**a**) the mean fluorescence normalized by the control fluorescence obtained by 2D-array (**b**) the mean fluorescence normalized by the control fluorescence obtained by FCM of cells exposed to increasing Cu and CuO-NPs concentrations.

Furthermore, the results obtained by the 2D-arrays were generally comparable with those obtained by FCM, however the differences between the treatments normalized by the control over the exposure time were more pronounced for the 2D-arrays especially for 10^−5^ M Cu (Figure 4a,b). Indeed, a significant decrease of the chlorophyll fluorescence was found for 10^−5^ M Cu at 120 min with 2D-array compared to the FCM. The mean fluorescence of 10^−5^ M Cu exposure normalized by the mean fluorescence of the control at 120 min decreased more than three times compared to 0 min exposure reaching 0.29 ± 0.06. Same exposure conditions measured by FCM showed only a decrease of 1.5 times from 0 min to 120 min to reach 0.65 ± 0.10. Similarities between FCM and DEP-based 2D- arrays present the capabilities of the last tool to be used for a rapid and non-invasive fluorescence detection for Cu and CuO-NPs sensing. The obtained results about the effect of the CuO-NPs on the algal fluorescence are consistent with the existing literature [24,25,26]. For example, the chlorophyll fluorescence emission measured by FCM decreased significantly only when *C. reinhardtii* cells were exposed to 100 and 1000 mg L^−1^ of CuO-NPs [26]. Another study using FCM showed an induction of the chlorophyll bleaching at a concentration of 10 mg L^−1^ in *C. reinhardtii* with the generation of reactive oxygen species (ROS) [24]. The results of the present work are also consistent with the results of previous studies where core–shell CuO-NPs induced ROS formation was responsible for a strong alteration of PSII photochemistry at concentrations from 0.01 g L^−1^ [25]. This decrease of the fluorescence obtained for CuO-NPs could be related to leaching of Cu ions from CuO-NPs [22]. Indeed a 24 h bleaching effect of 6 × 10^−6^ M Cu on the fluorescence of *C. reinhardtii* has already been shown with FCM [10]. Similarly, concentration range of Cu from 0.075 mg L^−1^ to 25 g L^−1^ reduced chlorophyll fluorescence in numerous microalgae such as *Pseudokirchneriella subcapitata* [27], *Ulva pertusa* [28], *P. tricornutum* [29], *C. eugametos* [7], *Scenedesmus incrassatulus* [30], and *Chlorella pyrenoidosa* [31].

Cu is known to induce an inhibition of the electron transfer in the chloroplast, inhibition of the production of the photosynthetic pigments, and damages to the membrane of the chloroplast [32,33,34,35]. For example, cells of *Chlamydomonas moewusii* and *Chlorella vulgaris* showed a decrease of the fluorescence of the cells as well as a disruption of the membrane integrity revealed by a fluorescent probe, propidium iodide, when they were exposed to 30 mg L^−1^ of copper. The decrease of chlorophyll fluorescent emission appeared in *C. moewusii* after 3 h of exposure to copper concentrations higher than 10 mg L^−1^ [35]. It was also shown that the decrease of the chlorophyll fluorescence was correlated to a change at the genomic level. Several genes or gene transcripts (i.e., *psb*D, *pet*D, *psaB*, *pet*F) related to respiration or photosynthesis underwent a reduction in their expression after 48 h of exposure to copper at concentrations ranging from 20 to 40 µM [36]. These results confirmed the role of copper in the inhibition of the electron transfer into the respiratory electron transport chains.

Furthermore, chlorophyll bleaching was observed by FCM when microalgae were exposed to high concentration of CuO-NPs [24,26] and core–shell CuO-NPs [25]. In contrary, FCM measurements of *Pseudo-nitzschia multiseries* and *Pseudo-nitzschia delicatissima* showed an increase of the chl *a* fluorescence during the first 72 h when they were exposed to Cu concentrations from 46 µg L^−1^ to 139 µg L^−1^ [37]. Same observations were made with *Microcystis aeruginosa* exposed for 24 h to intermediate concentration of Cu [38]. According to these literature results, the effects after Cu exposure on chl *a* fluorescence seem to be dependent to species, time, and concentration of Cu [37]. Despite high sensitive measurements reported with FCM in literature; in this study, the differences in the average fluorescence at 120 min were more pronounced for 2D-arrays than for FCM showing the good chlorophyll fluorescence detection of our tool in the tested concentration range of copper.

The use of 2D-array in our study offers some advantages compared to FCM. For example, the formation of 2D-cellular arrays facilitated fluorescence measurements by providing a larger cell measurement surface with a higher number of cells in comparison with no AC-field applied. The 2D-array tool could allow for a detection of multiple biological endpoints by combining stain-free chlorophyll fluorescence detection with specific fluorescent probe detection giving information on the cell membrane permeability or DNA damage. This multiple endpoints assessment is also achievable with FCM. However, the use of 2D-array allows the formation of a single layer of cells that can be then observed in a facile and reliable way for microscope analysis. This monolayer allows also a single cells image analysis compared to FCM. Thus, each cell can be observed under fluorescence microscopy and an image of each cell can be saved. Moreover, compared to other biosensors, 2D-array allows the immobilization of the cells without the use of chemical compounds and the disassembly of the membrane-like structure occurred when the electric field is switched off.

Because of these properties, 2D-array seems to be a suitable lab-on-chip tool for evaluating the mode of action of toxic contaminants without chemical immobilization or high amount of biological material [17]. These two different approaches, fluorescent microscopy and flow cytometry, were applied to detect chlorophyll fluorescence variation upon CuO-NPs and Cu exposure. In all cases, a decrease of the fluorescence was found but at different levels depending on the applied method. Several studies already conclude that chlorophyll fluorescence is a sensitive tool for a rapid detection of contaminants and/or environmental parameters with damaging effects on organisms capable of photosynthesis [35]. Indeed, a decrease of the fluorescence was shown for plant cells exposed to several harmful compounds by fluorescent microscopy [39,40] and flow cytometry monitoring [7,41,42].

### 3.3. 2D-Assembly Based Sensing of Fluorescence during Short-Term Exposure to Contaminants

**Figure 5:**
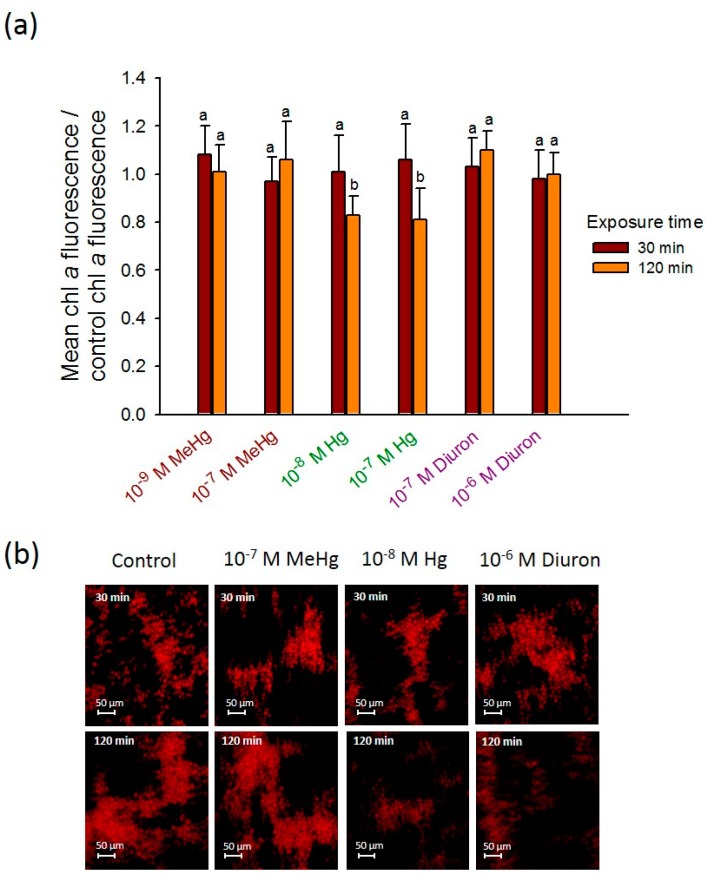
2D-array sensing of chlorophyll fluorescence during short-term exposure. (**a**) Fluorescence intensity for different chemicals; The different letters indicate the presence or absence of statistically significant differences (*p* < 0.05); (**b**) Example of fluorescence microscopy images of the chlorophyll of 2D-array by DEP. The cells in the formed non-uniform 2D-algal assembly of trapped cells after 30 or 120 min of exposure to contaminants.

To determine the applicability of 2D-whole cell arrays to probe the effect of other contaminants on *C. reinhardtii*, the changes in the chlorophyll fluorescence were followed during short-term exposure of 30 and 120 min to Hg, MeHg, and diuron (Figure 5). 120 min-exposure of 10^−8^ M Hg and 10^−6^ M diuron induced a small decrease in fluorescence where the values reached from 15.10 ± 1.90 a.u. and 14.60 ± 1.56 a.u. at 30 min to 12.7 ± 1.14 a.u. and 13.10 ± 1.22 a.u. at 120 min, respectively. These results are consistent with the ability of diuron to inhibit the quantum yield of PSII (Fv/Fm). Indeed, diuron exposure from 0.14 µM for 80 min to other green alga *Pseudokirchneriella subcapitata* showed to induce a suppression of Fv/Fm [43]. By contrast, both concentrations of MeHg and 10^−7^ M Hg showed no effect on the fluorescence of the cells after 30 and 120 min exposure as compared with the control (Figure 5). The results has also illustrated that the 2D-assembly of immobilized cells are non-uniformed (Figure 3b and Figure 5b). However, we do not believe this could affect the data analysis since we take into consideration the overall the area of the microfluidic chamber between the four point needle electrodes. As described in Section 2.2, the corrected fluorescence takes into account the 2D-cellular assembly and is normalized by the area to obtain the mean *CF* value per pixel.

Overall, the results obtained with this set of environmental contaminants demonstrated that at short-term exposure the chlorophyll fluorescence is less sensitive parameter when compared with the ROS biomarker [17]. Indeed, the decrease of the aquatic plant *Microsorium pteropus* or the brown seaweed *Hizikia fusiformis* chl-*a* content exposed to mercury was observed only at long exposure time e.g., 72 h to 33 µg L^−1^ Hg [44,45]. Same observations were made in *C. moewusii* exposed to 10^−7^ M MeHg [46]. Thus, DEP-based 2D-arrays for rapid chlorophyll change detection seems to be suitable tool for mostly probing the effect of very high concentrations of contaminant, as Cu and CuO-NPs. Otherwise a longer period of exposure has to be applied in order to detect a measureable change.

## 4. Conclusions

The results and their analysis confirm that AC-dielectrophoresis allows rapid algal assembly in planar 2D whole-cell array with no alteration of the fluorescence of the cells. 2D-array was used to follow in a non-invasive way the short-term effect of high concentrations of copper and copper oxide nanoparticles following the changes in the chlorophyll fluorescence of the cells with microscopy, which does not need the addition of any stain. The data showed a significant decrease of the chlorophyll fluorescence when *C. reinhardtii* was exposed to high concentrations of CuO-NPs and Cu; results which are consistent with the flow cytometry measurements used as a comparative method. The tool was also applied to explore the short-term effect of Hg, MeHg, and diuron on the chlorophyll fluorescence, where the statistically significant effects were observed only for Hg. Overall, the 2D-cellular array on a chip was verified as a tool for facile determination of the effect of high concentrations of important environmental contaminants. 

## References

[1] Maxwell K., Johnson G.N. (2000). Chlorophyll fluorescence—A practical guide. J. Exp. Bot..

[2] Rohacek K., Bartak M. (1999). Technique of the modulated chlorophyll fluorescence: Basic concepts, useful parameters, and some applications. Photosynthetica.

[3] Kumar K.S., Dahms H.U., Lee J.S., Kim H.C., Lee W.C., Shin K.H. (2014). Algal photosynthetic responses to toxic metals and herbicides assessed by chlorophyll a fluorescence. Ecotoxicol. Environ. Saf..

[4] Buonasera K., Lambreva M., Rea G., Touloupakis E., Giardi M.T. (2011). Technological applications of chlorophyll a fluorescence for the assessment of environmental pollutants. Anal. Bioanal. Chem..

[5] Brayner R., Coute A., Livage J., Perrette C., Sicard C. (2011). Micro-algal biosensors. Anal. Bioanal. Chem..

[6] Frense D., Muller A., Beckmann D. (1998). Detection of environmental pollutants using optical biosensor with immobilized algae cells. Sens. Actuators B Chem..

[7] Franqueira D., Orosa M., Torres E., Herrero C., Cid A. (2000). Potential use of flow cytometry in toxicity studies with microalgae. Sci. Total Environ..

[8] Jamers A., Blust R., De Coen W., Griffin J.L., Jones O.A.H. (2013). Copper toxicity in the microalga *Chlamydomonas reinhardtii:* An integrated approach. Biometals.

[9] Stauber J.L., Franklin N.M., Adams M.S. (2002). Applications of flow cytometry to ecotoxicity testing using microalgae. Trends Biotechnol..

[10] Ahmed N.B., Masse S., Laurent G., Piquemal J.Y., Yéprémian C., Brayner R., Coradin T. (2017). Optical microalgal biosensors for aqueous contaminants using organically doped silica as cellular hosts. Anal. Bioanal. Chem..

[11] Suarez G., Santschi C., Martin O.J.F., Slaveykova V.I. (2013). Biosensor based on chemically-designed anchorable cytochrome *c* for the detection of H_2_O_2_ released by aquatic cells. Biosens. Bioelectron..

[12] Putzbach W., Ronkainen N.J. (2013). Immobilization techniques in the fabrication of nanomaterial-based electrochemical biosensors: A review. Sensors.

[13] Koman V.B., Santschi C., von Moos N.R., Slaveykova V.I., Martin O.J.F. (2015). Portable oxidative stress sensor: Dynamic and non-invasive measurements of extracellular H_2_O_2_ released by algae. Biosens. Bioelectron..

[14] Suscillon C., Velev O.D., Slaveykova V.I. (2013). Alternating current-dielectrophoresis driven on-chip collection and chaining of green microalgae in freshwaters. Biomicrofluidics.

[15] Bakewell D.J., Bailey J., Holmes D. (2013). Advancing image quantification methods and tools for analysis of nanoparticle electrokinetics. AIP Adv..

[16] Chuang C.H., Ju J.W., Huang Y.W. (2013). Enhancing fluorescent response of immunosensing by a dielectrophoresis chip with transparent electrodes and microcavities array. IET Micro Nano Lett..

[17] Siebman C., Velev O.D., Slaveykova V.I. (2015). Two-dimensional algal collection and assembly by combining ac-dielectrophoresis with fluorescence detection for contaminant-induced oxidative stress sensing. Biosensors.

[18] Waters J.C. (2009). Accuracy and precision in quantitative fluorescence microscopy. J. Cell Biol..

[19] Gupta S., Alargova R.G., Kilpatrick P.K., Velev O.D. (2010). On-chip dielectrophoretic coassembly of live cells and particles into responsive biomaterials. Langmuir.

[20] Gray D.S., Tan J.L., Voldman J., Chen C.S. (2004). Dielectrophoretic registration of living cells to a microelectrode array. Biosens. Bioelectron..

[21] Graham D.M., Messerli M.A., Pethig R. (2012). Spatial manipulation of cells and organelles using single electrode dielectrophoresis. Biotechniques.

[22] Ivask A., Bondarenko O., Jepihhina N., Kahru A. (2010). Profiling of the reactive oxygen species-related ecotoxicity of CuO, ZnO, TiO_2_, silver and fullerene nanoparticles using a set of recombinant luminescent *Escherichia coli* strains: Differentiating the impact of particles and solubilised metals. Anal. Bioanal. Chem..

[23] Von Moos N., Bowen P., Slaveykova V.I. (2014). Bioavailability of inorganic nanoparticles to planktonic bacteria and aquatic microalgae in freshwater. Environ. Sci. Nano.

[24] Von Moos N., Maillard L., Slaveykova V.I. (2015). Dynamics of sub-lethal effects of nano-cuo on the microalga *Chlamydomonas reinhardtii* during short-term exposure. Aquat. Toxicol..

[25] Saison C., Perreault F., Daigle J.C., Fortin C., Claverie J., Morin M., Popovic R. (2010). Effect of core-shell copper oxide nanoparticles on cell culture morphology and photosynthesis (photosystem II energy distribution) in the green alga, *Chlamydomonas reinhardtii*. Aquat. Toxicol..

[26] Melegari S.P., Perreault F., Costa R.H.R., Popovic R., Matias W.G. (2013). Evaluation of toxicity and oxidative stress induced by copper oxide nanoparticles in the green alga *Chlamydomonas reinhardtii*. Aquat. Toxicol..

[27] Soto P., Gaete H., Hidalgo M.E. (2011). Assessment of catalase activity, lipid peroxidation, chlorophyll-a, and growth rate in the freshwater green algae *Pseudokirchneriella subcapitata* exposed to copper and zinc. Lat. Am. J. Aquat. Res..

[28] Han T., Kang S.H., Park J.S., Lee H.K., Brown M.T. (2008). Physiological responses of *Ulva pertusa* and U-armoricana to copper exposure. Aquat. Toxicol..

[29] Chen H., Chen J., Guo Y., Wen Y., Liu J., Liu W. (2012). Evaluation of the role of the glutathione redox cycle in Cu(II) toxicity to green algae by a chiral perturbation approach. Aquat. Toxicol..

[30] Perales-Vela H.V., Gonzalez-Moreno S., Montes-Horcasitas C., Canizares-Villanueva R.O. (2007). Growth, photosynthetic and respiratory responses to sub-lethal copper concentrations in *Scenedesmus incrassatulus* (*Chlorophyceae*). Chemosphere.

[31] Zhou G.J., Peng F.Q., Zhang L.J., Ying G.G. (2012). Biosorption of zinc and copper from aqueous solutions by two freshwater green microalgae *Chlorella pyrenoidosa* and *Scenedesmus obliquus*. Environ. Sci. Pollut. Res. Int..

[32] Sabatini S.E., Juárez A.B., Eppis M.R., Bianchi L., Luquet C.M., Ríos de Molina Mdel C. (2009). Oxidative stress and antioxidant defenses in two green microalgae exposed to copper. Ecotoxicol. Environ. Saf..

[33] Yan H., Pan G. (2002). Toxicity and bioaccumulation of copper in three green microalgal species. Chemosphere.

[34] Charles A.L., Markich S.J., Ralph P. (2006). Toxicity of uranium and copper individually, and in combination, to a tropical freshwater macrophyte (*Lemna aequinoctialis*). Chemosphere.

[35] Prado R., Garcia R., Rioboo C., Herrero C., Cid A. (2015). Suitability of cytotoxicity endpoints and test microalgal species to disclose the toxic effect of common aquatic pollutants. Ecotoxicol. Environ. Saf..

[36] Wei Y., Zhu N., Lavoie M., Wang J., Qian H., Fu Z. (2014). Copper toxicity to *Phaeodactylum tricornutum*: A survey of the sensitivity of various toxicity endpoints at the physiological, biochemical, molecular and structural levels. Biometals.

[37] Lelong A., Jolley D.F., Soudant P., Hegaret H. (2012). Impact of copper exposure on *Pseudo-nitzschia* spp. Physiology and domoic acid production. Aquat. Toxicol..

[38] Hadjoudja S., Vignoles C., Deluchat V., Lenain J.F., Le Jeune A.H., Baudu M. (2009). Short term copper toxicity on *Microcystis aeruginosa* and *Chlorella vulgaris* using flow cytometry. Aquat. Toxicol..

[39] Hjorth M., Mondolot L., Buatois B., Andary C., Rapior S., Kudsk P., Mathiassen S.K., Ravn H.W. (2006). An easy and rapid method using microscopy to determine herbicide effects in *Poaceae weed* species. Pest Manag. Sci..

[40] Nancharaiah Y.V., Rajadurai M., Venugopalan V.P. (2007). Single cell level microalgal ecotoxicity assessment by confocal microscopy and digital image analysis. Environ. Sci. Technol..

[41] Gonzalez-Barreiro O., Rioboo C., Cid A., Herrero C. (2004). Atrazine-induced chlorosis in *Synechococcus elongatus* cells. Arch. Environ. Contam. Toxicol..

[42] Prado R., Rioboo C., Herrero C., Suarez-Bregua P., Cid A. (2012). Flow cytometric analysis to evaluate physiological alterations in herbicide-exposed *Chlamydomonas moewusii* cells. Ecotoxicology.

[43] Choi H.Y., Hyun Y.C., Min Y.C., Min S.K. (2011). Effects of rapid thermal annealing on the optical properties of GaAs quantum dots grown by using the droplet epitaxy method. J. Korean Phys. Soc..

[44] Deng C.N., Zhang D.Y., Pan X.L., Chang F.Q., Wang S.Z. (2013). Toxic effects of mercury on PSI and PSII activities, membrane potential and transthylakoid proton gradient in *Microsorium pteropus*. J. Photochem. Photobiol. B.

[45] Zhu X.F., Zou D., Huang Y., Cao J., Sheng G., Wang G. (2015). Physiological responses of *Hizikia fusiformis* (*Phaeophyta*) to mercury exposure. Bot. Mar..

[46] Protopopov F.F., Matorin D.N., Seifullina N.K., Bratkovskaya L.B., Zayadan B.K. (2015). Effect of methylmercury on the light dependence fluorescence parameters in a green alga *Chlamydomonas moewusii*. Microbiology.

